# Transcriptional Interplay between *Malassezia restricta* and *Staphylococcus* Species Co-Existing in the Skin Environment

**DOI:** 10.4014/jmb.2212.12026

**Published:** 2023-01-06

**Authors:** Hyun Oh Yang, Yong-Joon Cho, Jae Min Lee, Kyoung-Dong Kim

**Affiliations:** 1 Department of Systems Biotechnology, Chung-Ang University, Anseong 17546, Republic of Korea; 2 Department of Molecular Bioscience, Kangwon National University, Chuncheon 24341, Republic of Korea

**Keywords:** *Malassezia*, *Staphylococcus*, aspartyl protease, biofilms, skin, pH

## Abstract

*Malassezia* and *Staphylococcus* are the most dominant genera in human skin microbiome. To explore the inter-kingdom interactions between the two genera, we examined the transcriptional changes in *Malassezia* and *Staphylococcus* species induced upon co-culturing. RNA-seq analyses revealed that genes encoding ribosomal proteins were upregulated, while those encoding aspartyl proteases were downregulated in *M. restricta* after co-culturing with *Staphylococcus* species. We identified MRET_3770 as a major secretory aspartyl protease coding gene in *M. restricta* through pepstatin-A affinity chromatography followed by mass spectrometry and found that the expression of MRET_3770 was significantly repressed upon co-culturing with *Staphylococcus* species or by incubation in media with reduced pH. Moreover, biofilm formation by *Staphylococcus aureus* was inhibited in the spent medium of *M. restricta*, suggesting that biomolecules secreted by *M. restricta* such as secretory aspartyl proteases may degrade the biofilm structure. We also examined the transcriptional changes in *S. aureus* co-cultured with *M. restricta* and found co-cultured *S. aureus* showed increased expression of genes encoding ribosomal proteins and downregulation of those involved in riboflavin metabolism. These transcriptome data of co-cultured fungal and bacterial species demonstrate a dynamic interplay between the two co-existing genera.

## Introduction

Microorganisms interact with each other either directly through physical contact or indirectly via chemical interactions in various ecosystems, such as microbial biofilms [1, 2], respiratory tract [3, 4], soil [5, 6], and plants [7, 8]. For example, *Malassezia* and *Candida*, when co-cultured, produce stronger biofilms resulting in increased resistance against antifungal reagents [2]. In the soil, physical interaction of actinomycetes with *Aspergillus* mycelia induces the expression of a gene cluster associated with archetypal polyketides, suggesting that direct physical interactions contribute to the communication among microorganisms and induce gene expression [6]. In plant microbiome, a phenazine produced by the bacterium *Pseudomonas piscium* inhibits the activity of an acetyltransferase expressed by the fungal species *Fusarisu graminearum*, resulting in reduced acetylation and repression of gene expression in the fungal pathogen. Thus, the epigenome of a fungal pathogen could be influenced by a chemical produced by co-existing bacteria [8].

The skin is the largest organ acting as the primary physical barrier against the invasion of pathogens and external substances from the environment [9, 10]. Skin is inhabited by a large variety of microorganisms, and while some of these microorganisms are harmless or even helpful to the host, pathogenic microorganisms also coexist [10, 11]. The microbial ecosystem consisting of multiple species coexisting in the same habitat is referred as the microbiome. With the development of metagenome analysis and next-generation sequencing, it has been possible to identify the members of the microbiome present in the skin [12]. Skin microbiota is composed of bacteria, fungi, and viruses. While the distribution of bacterial species differs throughout the body, *Cutibacterium*, *Corynebacterium*, and *Staphylococcus* are generally the dominant genera [13, 14]. *Malassezia* is the dominant fungal genus in almost all regions of the skin except the feet, which is colonized by diverse types of fungi [13, 15].

*Malassezia* species are known to exist in oily skin environments since they cannot synthesize fatty acids and should acquire them externally. Thus, *Malassezia* species secrete multiple lipases involved in lipid metabolism and pathogenicity [16]. Similarly, proteases, important hydrolytic enzymes for *Malassezia*, are also secreted and used to utilize the nutrients present in the skin environment [17]. Proteases are classified into multiple types, including serine, aspartyl, and metalloprotease; at least one type of protease is expressed by each *Malassezia* species [17]. In previous works, predominant secreted aspartyl proteases (SAP) were identified in *Malassezia globosa* and *Malassezia furfur* [18, 19]. The SAP in *M. globosa* inhibited the formation of biofilm in *S. aureus* and the SAP in *M. furfur* had a wide range of substrates including extracellular matrix [18, 19]. *Malassezia restricta* expresses a total of 17 secreted proteases, 12 of which are aspartyl proteases [17], but the predominant SAP and the role of SAP have not been identified in *M. restricta*.

In the present work, we studied the interplay between the fungus *M. restricta* and two bacterial species of the genus *Staphylococcus*, which are predominant in skin microbiome. We comprehensively examined transcriptional changes in *M. restricta* and *S. aureus* upon co-culturing. We found that the expression levels of ribosomal protein-encoding genes (RPGs) were upregulated, while SAP-encoding genes were downregulated in *M. restricta* co-cultured with *Staphylococcus* species. We identified the predominant SAP, MRET_3770, in *M. restricta* and noticed that MRET_3770 was transcriptionally repressed by co-culturing with *Staphylococcus* species and by lower pH levels. In bacterial RNA-seq, we observed upregulation of *Staphylococcus* RPGs and downregulation of genes involved in riboflavin metabolism in co-cultured *S. aureus*.

## Materials and Methods

### Strains and Growth Conditions

*M. restricta* KCTC 27527, *Staphylococcus epidermidis* KCTC 13172, and *Staphylococcus aureus* NCTC 8325 were obtained from the Korean Collection for Type Cultures (KCTC, Korea). *M. restricta* KCTC 27527 was maintained in modified Dixon (mDixon) medium (3.6% malt extract [w/v], 2% bile salt [w/v], 1% tween 40, 0.6%peptone [w/v], 0.2% glycerol, and 0.2% oleic acid) [34, 35]. Lower pH mDixon medium was adjusted to pH 5.0 or 5.5 with 5 N HCl. The *Staphylococcus* cells were maintained in tryptic soy broth (TSB; 1.7% casein peptone, 0.5%sodium chloride, 0.3% soybean peptone, 0.25% glucose, and 0.25% dipotassium phosphate) [36]. For the co-culture, *M. restricta* (OD600: 1) and *S. epidermidis* or *S. aureus* (OD600: 0.1) were inoculated in mDixon medium and co-cultured at 34°C for 12 h with shaking. To prepare bacterial spent medium, *S. epidermidis* or *S. aureus* (OD600: 0.1) cells were inoculated in mDixon medium and cultured at 34°C for 12 h. Then, the bacterial spent medium was separated from bacterial cells by centrifugation. Similarly, *M. restricta* (OD600: 1) cells were cultured for 12 h, and the *M. restricta* spent medium was separated by centrifugation.

### Enrichment of Secretory Aspartyl Protease Using Pepstatin A-Agarose Resin

Aspartyl proteases were enriched from the extracellular culture media using pepstatin A-agarose resin (Goldbio, USA). Briefly, prewashed pepstatin A-agarose resins were incubated with the concentrated extracellular media with shaking at 4°C for 1 h. The resins were collected into column with binding buffer (0.1 M citrate and 0.5 M sodium chloride at pH 3.0) and subsequently washed with 0.5 M sodium chloride at pH 3.0 and eluted with elution buffer (0.1 M sodium bicarbonate and 0.5 M sodium chloride at pH 8.7).

### Crystal Violet Assay

Diluted cells were seeded and incubated on 96-well cell culture plates (SPL, Korea) for 12 h. The culture supernatant was removed, and the cells were washed with distilled water (DW) to remove planktonic microbial cells. After the plate was dried, 0.1% crystal violet was added to stain the bacterial biofilm firmly attached to the plate. After 10 min, the plate was washed with DW, and non-specific dye was decolorized using 30% acetic acid. The crystal violet staining was measured using spectrophotometry at OD600.

### mRNA Sequencing and Analysis

Total RNA from two biological replicates for each condition was processed to deplete the ribosomal RNA using a NEBNext rRNA Depletion Kit (Bacteria; #E7850S, NEB). Sequencing libraries for RNA-Seq were constructed using TruSeq Stranded mRNA Library Prep (48 Samples; #20020594, Illumina) following the manufacturer’s instructions. Sequencing was performed using a NovaSeq 6000 instrument (Illumina), following the manufacturer’s protocol, which generated 101-bp-long paired-end reads. The sequencing adapter removal and quality-based trimming of the raw data were performed using Trimmomatic v.0.36 [37]. Cleaned reads were mapped to the reference genome (NCBI assembly; GCF_003290485.1 for *M. restricta* KCTC 27525 and GCA_026314335.1 for *S. epidermidis* KCTC 13172) using hisat2 v.2.2.1 [38]. We used the parameter ‘-k 1’ in mapping and added the option ‘--no-spliced-alignment’. For counting the reads mapped to each coding sequence (CDS), featureCounts was used [39]. Finally, the counts from each CDS were normalized using the DeSeq2 package [40].

## Results

### Transcriptional Changes in *Malassezia* Gene Expression upon Co-Culturing with *S. epidermidis* or *S. aureus*

**Fig. 1:**
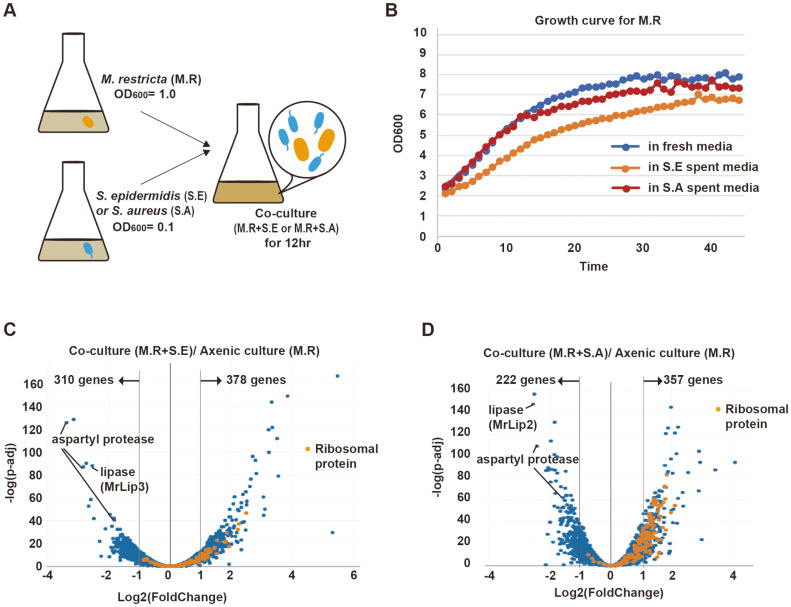
Transcriptional changes in *M. restricta* co-cultured with *Staphylococcus* spp. **A**. Co-culture of *M. restricta* (M.R) and *Staphylococcus* species. *M. restricta* (OD600, 1.0) and *Staphylococcus* species (OD600, 0.1) were inoculated in mDixon media and co-cultured for 12 h. **B**. Growth curves of *M. restricta* in bacteria spent media. Blue line indicates *M. restricta* in fresh media, yellow line indicates *M. restricta* (M.R) in *S. epidermidis* (S.E) spent media, and orange line indicates *M. restricta* in *S. aureus* (S.A) spent media. **C, D**. The volcano plots of fungal transcriptional changes after co-culturing with *S. epidermidis* (S.E) (C) and *S. aureus* (S.A) (D). The orange dots indicate genes encoding ribosomal proteins.

To investigate the functional interactions between *M. restricta* and *Staphylococcus* species, *M. restricta* (OD600: 1.0) and *Staphylococcus* species (OD600: 0.1) were inoculated in mDixon media and co-cultured for 12 h (Fig. 1A). As the growth rates of *Staphylococcus* species are higher than that of *M. restricta*, *M. restricta* with a higher OD600 (ten-fold higher than that of *Staphylococcus* species) were inoculated at the initial step of co-culturing. We tried to measure the growth rate of *M. restricta* in the co-culturing medium using a hemocytometer, but the number of colonies could not be detected due to the preferential aggregation of *M. restricta* cells. Instead, the growth rate of *M. restricta* in the co-culture was indirectly measured by using bacterial spent medium through the personal bioreactor (RTS-1). To prepare bacterial spent medium, *Staphylococcus* cells (OD600: 0.1) were inoculated in mDixon medium and cultured for 12 h. Then, the bacterial spent medium was separated from the bacteria by centrifugation. Incubation of bacterial species in bacterial spent medium mimics co-culturing conditions. The growth rate of *M. restricta* was slightly lower in *S. aureus* spent medium and further decreased in *S. epidermidis* spent medium (Fig. 1B).

Next, the expressional changes in *Malassezia* genes upon co-culturing with *S. aureus* or *S. epidermidis* were examined by RNA-seq analysis. We found that, co-culturing with *S. epidermidis* resulted in an increase (at least two-fold) in the expression levels of 378 genes and downregulation of 310 genes in *M. restricta* (Fig. 1C). Similarly, co-culturing with *S. aureus* induced the upregulation of 357 and downregulation of 222 genes in *M. restricta* (Fig. 1D). Based on gene ontology (GO) analysis, GO groups related to transcription and translation were significantly upregulated in both co-culturing conditions (Figs. S1A and S1C). On the contrary, GO groups related to lipase and aspartyl protease were significantly downregulated (Figs. S1B and S1D).

**Fig. 2:**
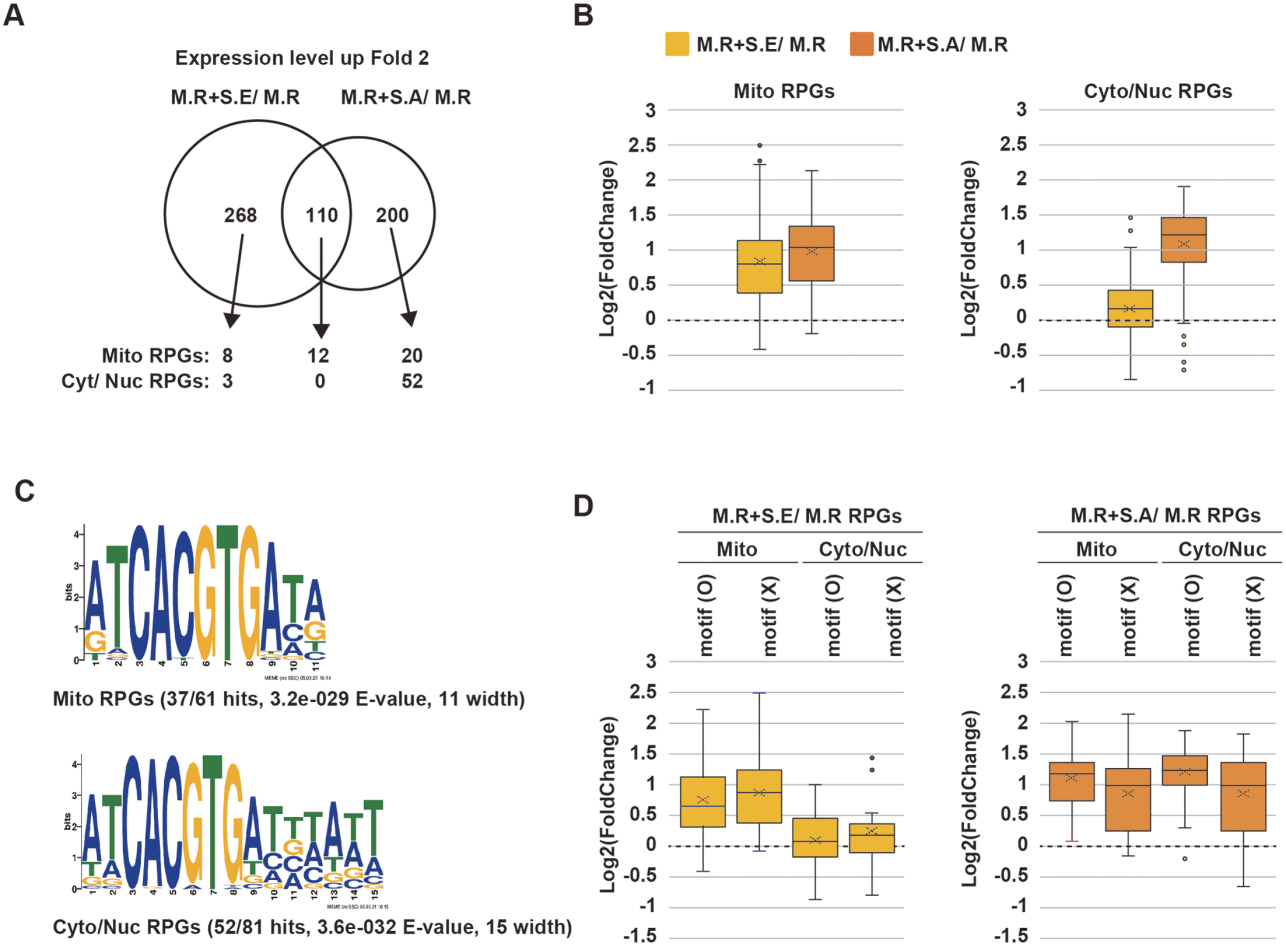
Upregulation of ribosomal proteins in *M. restricta* upon co-culturing with *Staphylococcus* spp. **A**. Venn diagram showing the numbers of genes exhibiting more than 2-fold increase in *M. restricta* (M.R) upon co-culturing compared to axenic culture. Black arrows point to mitochondrial ribosomal protein-encoding genes (mito RPGs) and cytoplasmic/ nuclear ribosomal protein-encoding genes (cyt/nuc RPGs). **B**. Box plots showing the expressional changes in mito RPGs and in cyto/nuc RPGs in *M. restricta* co-cultured with *S. epidermidis* compared to *M. restricta* axenic culture (M.R+S.E/ M.R) and in *M. restricta* co-cultured with *S. aureus* compared to *M. restricta* axenic culture (M.R+S.A/ M.R). **C**. Motif sequences of identified mito and cyto/nuc RPGs analyzed by MEME Suite 5.5.0. **D**. Box plots showing the expressional changes in mito RPGs and cyto/nuc RPGs according to the presence of the motif sequence.

Induction of Ribosomal Protein-Coding Genes in *Malassezia* upon Co-Culturing with *Staphylococcus* Species The expression levels of ribosomal protein-encoding genes (RPGs) in *M. restricta* were noticeably elevated after co-culturing with *Staphylococcus* species, especially with *S. aureus* (Figs. 1C and 1D). Among the total 137 annotated *Malassezia* RPGs, 12 RPGs were commonly induced by co-culturing with either *Staphylococcus* species, and 72 RPGs were only induced by co-culturing with *S. aureus* (Fig. 2A). When *M. restricta* was co-cultured with *S. aureus*, 84 RPGs (out of 137; 61.3%) were upregulated more than two-fold compared to *M. restricta* axenic culture (Fig. 2A). We noticed that mitochondrial RPGs (Mito RPGs) tended to be upregulated in both co-culture conditions, but cytosolic and nuclear RPGs (Cyto/Nuc RPGs) were greatly upregulated in *M. restricta* co-cultured with *S. aureus* but not in *M. restricta* co-cultured with *S. epidermidis* (Fig. 2B). Thus, expression levels of mitochondrial and cytosolic/nuclear RPGs are differentially regulated, and the two *Staphylococcus* species have distinct effects on the regulation of *Malassezia* ribosomal proteins.

To investigate the molecular mechanisms underlying the differential regulation of Cyto/Nuc RPGs and Mito RPGs in *M. restricta*, the motif sequences on the promoter regions of the RPGs were examined. We found that approximately 60% of the RPGs possessed the core motif sequence ‘ATCACGTGA’ and that the motif sequences did not clearly differ between Cyto/Nuc RPGs and Mito RPGs. However, the promoter regions of Cyto/Nuc RPGs had the extended consensus sequence ‘ATCACGTGATTTATT’, which may alter the binding affinities of regulatory proteins (Fig. 2C). Next, we examined the expression levels of the RPGs according to the presence of the identified motif and found that the expression of RPGs with the motif were significantly upregulated in *M. restricta* co-cultured with *S. aureus*, but not in that co-cultured with *S. epidermidis* (Fig. 2D).

Taken together, we found that Cyto/Nuc RPGs were upregulated in *M. restricta* co-cultured with either of the two *Staphylococcus* species, especially with *S. aureus*. Moreover, our observations suggest that the extended motif sequences on the promoter regions of Cyto/Nuc RPGs in *M. restricta* might have a role in the upregulation of the genes.

### Downregulation of Major Aspartyl Proteases in *M. restricta* upon Co-Culturing with *Staphylococcus* Species

We observed that the genes encoding aspartyl proteases and lipases, which are important for *M. restricta* to utilize the nutrients available in host skin, were significantly downregulated in *M. restricta* after co-culturing with *Staphylococcus* species (Figs. 1C and 1D, S1C). First, we summarized the changes in the expression levels of lipase annotated genes in *M. restricta* (Figs. S2A and S2B). MrLIP3 and MrLIP2 were significantly downregulated in *M. restricta* after co-culturing with *S. epidermidis* and *S. aureus*, respectively (Figs. 1C and 1D, S2A). However, the transcriptional expression of MrLIP1, which is the lipase with the highest expression levels in *M. restricta*, was not downregulated by co-culturing with *Staphylococcus* species; instead, it was slightly upregulated upon co-culturing with *S. epidermidis*.

Of the 79 genes downregulated in both co-culturing conditions, three genes encoding aspartyl proteases, MRET_3770, MRET_2827, and MRET_4390, were markedly downregulated (Figs. 2A and 2B). We summarized the changes in the expression levels of genes annotated as aspartyl proteases in *M. restricta* (Fig. 2B). Interestingly, MRET_3770 showed the highest levels of expression in *M. restricta* cultured alone; however, it exhibited the strongest downregulation in both co-culturing conditions. MRET_2827 and MRET_4390 also showed relatively higher expression levels and were significantly downregulated in both co-culturing conditions (Figs. 2B and 2C). RT-qPCR analysis confirmed that the expression levels of MRET_3770, MRET_2827, and MRET_4390 were significantly decreased in co-cultures with *Staphylococcus* species, except in the case of MRET_4390 in the co-culture with *S. aureus*. However, the expression levels of other aspartyl proteases, such as MRET_2886 and MRET_2661, were not altered (Fig. 2D). Taken together, the mainly expressed aspartyl proteases, but not lipases, in *M. restricta* are transcriptionally repressed by *Staphylococcus* species.

### Identification of the Major Secretory Aspartyl Proteases in *M. restricta*

**Fig. 4:**
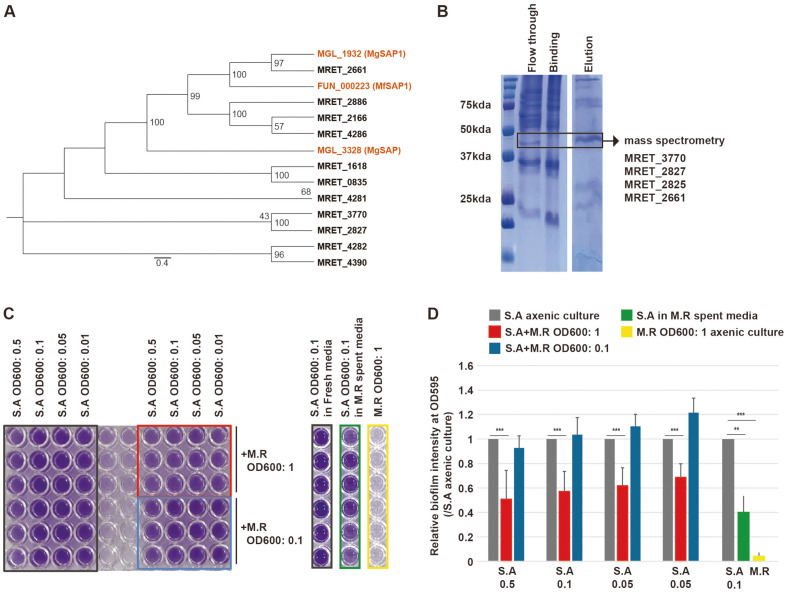
Identification of mainly expressed secretory aspartyl proteases in *M. restricta* and the inhibitory effect of *M. restricta* on the formation of *S. aureus* biofilm. **A**. Unrooted phylogenetic tree of the putative secretory aspartyl proteases in *M. restricta* compared with MGL_1932 (MgSAP1) and MGL_3328 in *M. globosa* and FUN_000223 (MfSAP1) in *M. furfur*. The numbers at the nodes indicate the bootstrap values. **B**. Coomassie brilliant blue staining of SDSPAGE of the extracellular media (Flow though), binding buffer (Binding), and the enriched fraction (Elution) from pepstatin A-agarose affinity purification. The contents of the band in the block box (approximately 42 kDa) were analyzed by orbitrap mass spectrometry and identified as MRET_3770, MRET_2827, MRET_2825, and MRET_2661. **C**. Biofilm enrichment was measured by the crystal violet assay as described in the Methods section. Serial concentration of *S. aureus* (S.A) in axenic culture, S.A co-cultured with *M. restricta* (M.R), or S.A cultured in M.R spent media were examined. Black box, S.A axenic culture; red box, S.A co-cultured with M.R (OD600: 1); blue box, S.A co-cultured with M.R (OD600: 0.1); green box, S.A cultured in M.R spent media; yellow box, M.R axenic culture. **D**. The ratios of biofilm formation. Biofilm formation was measured by spectrophotometry. *, *p* < 0.1; **, *p* < 0.01; ***, *p* < 0.001.

To identify the major secretory aspartyl proteases (SAP) in *M. restricta*, we listed the genes annotated as SAP using the MEROPS and SignalP 5.0. We noted that *M. restricta* had a total of 13 genes encoding putative aspartyl proteases, of which, 11 were secretory (Fig. S3A). Genes encoding other types of secretory proteases were also present, including metalloproteases (four genes), serine proteases-(seven genes), and threonine protease (one gene) (Fig. S3A). To determine the major SAP in *M. restricta*, an alignment tree of the 11 annotated SAPs were created comparing them to the SAPs in *M. globosa* (MGL_1932) and *M. furfur* (FUN_000223) that were previously identified as functional SAPs (Fig. 4A). MRET_2661 was identified as the closest to the SAPs of *M. globosa* (MGL_1932) and of *M. furfur* (FUN_000223) [18].

Next, the SAPs present in the culture media were captured by affinity chromatography using pepstatin A-agarose resin, and the proteins were visualized on SDS-PAGE (Fig. 4B). Then, the captured proteins were identified through the MALDI-TOF analysis (Fig. 4B). MRET_3770 and MRET_2827, which exhibited the highest expression levels, were identified as highly scored purified proteins, followed by MRET_2825 and MRET_2661 (Figs. 4B, S3B). Collectively, although not closely related to previously identified SAPs in other *Malassezia* species, MRET_3770 and MRET_2827 were the most abundant putative SAPs in *M. restricta*.

SAPs of *M. globosa* are known to inhibit the formation of the biofilm generated by *S. aureus* [19]. To investigate the inhibition of biofilm formation by *M. restricta*, we performed a crystal violet assay in both axenic and co-culture conditions. *S. aureus* at OD600 of 0.5, 0.1, 0.05, or 0.01 were co-cultured with *M. restricta* at OD600 of 1 or 0.1 on 96-well plates. After 12 h, the amounts of biofilm formed under axenic and co-culture conditions were quantitatively measured through the density of crystal violet. Biofilm formation by *S. aureus* was significantly reduced upon co-culturing with *M. restricta* at OD600 of 1, but not altered after co-culturing with *M. restricta* at OD600 of 0.1 (Figs. 4C and 4D), indicating that sufficient concentrations of *M. restricta* could inhibit the biofilm formation by *S. aureus*.

### Differential Expression of Putative SAP Genes at Lower pH

**Fig. 5:**
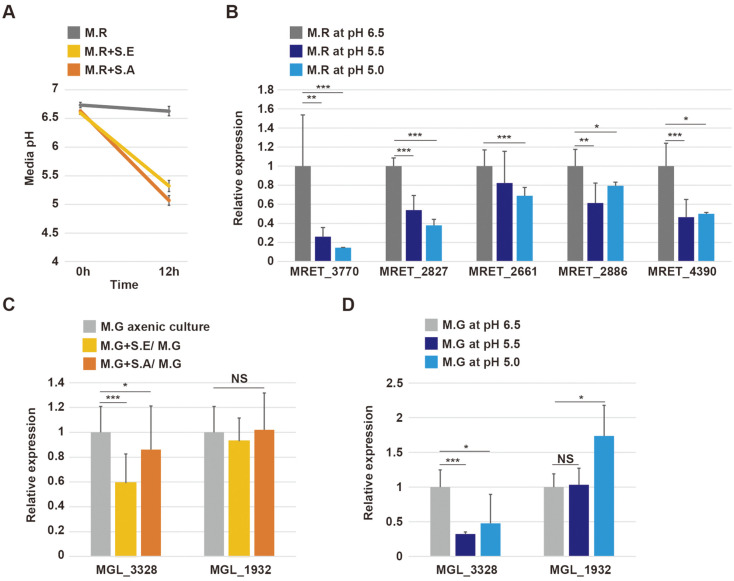
Transcriptional expression of secretory aspartyl proteases in *M. restricta* and *M. globosa* at lower pH. **A**. The pH of the media of *M. restricta* axenic culture (M.R), *M. restricta* co-cultured with *S. epidermidis* (M.R+S.E), and *M. restricta* co-cultured with *S. aureus* (M.R+S.A) before and after 12 h of incubation. The initial pH of the media was 6.5–6.7. **B**. Relative expression levels of genes encoding aspartyl proteases in *M. restricta* in media at pH 6.5 (M.R at pH 6.5), pH 5.5 (M.R at pH 5.5), and pH 5.0 (M.R at pH 5.0). Expression levels were normalized to those of actin (MRET_1518) and compared to the expression levels observed in M.R at pH 6.5. **C**. Relative expression levels of MGL_3328 and MGL_1932 in *M. globosa* (M.G) axenic culture, *M. globosa* co-cultured with *S. epidermidis* (M.G+S.E), and *M. globosa* co-cultured with *S. aureus* (M.G+S.A). Gene expression levels were normalized to those of actin (MGL_1986) and compared to the expression levels observed in M.G axenic culture. **D**. Relative expression of genes encoding aspartyl proteases in *M. globosa* at pH 6.5 (M.G at pH 6.5), pH 5.5 (M.G at pH 5.5), and pH 5.0 (M.G at pH 5.0). Gene expression levels were normalized to those of actin (MGL_1986) and compared to the expression levels observed in M.G at pH 6.5. *, *p* < 0.1; **, *p* < 0.01; ***, *p* < 0.001.

We noticed that the pH of the culture medium decreased from 6.5–6.8 to 5.0–5.5 after co-culturing with *Staphylococcus* species, while the pH of *M. restricta* axenic culture medium was maintained at around 6.5 (Fig. 5A). Accordingly, we examined the impact of reduced pH on the expression of SAP genes. The result of RT-qPCR indicated that the mRNA levels of putative SAPs including MRET_3770, MRET_2827, MRET_4390, MRET_2661 and MRET_2886 were significantly reduced in culture medium at pH 5.0–5.5 compared to culture medium at pH 6.0 (Fig. 5B), indicating that lower pH could result in the inhibition of the expression of SAP genes in *M. restricta*.

Next, we examined the effect of the pH of the culture media on the regulation of SAP genes in *M. globosa*, which were characterized in a previous study [19]. The expression of the previously characterized SAP gene MGL_1932 was not altered by co-culturing with either *Staphylococcus* species or by culturing in media with a lower pH, but the expression of MGL_3328, which is the SAP the highest expression levels in *M. globosa*, was reduced by co-culturing, especially with *S. epidermidis*, and in response to a decrease in pH (Figs. 5C and 5D).

### Changes in *Staphylococcus* Gene Expression upon Co-Culturing with *M. restricta*

**Fig. 6:**
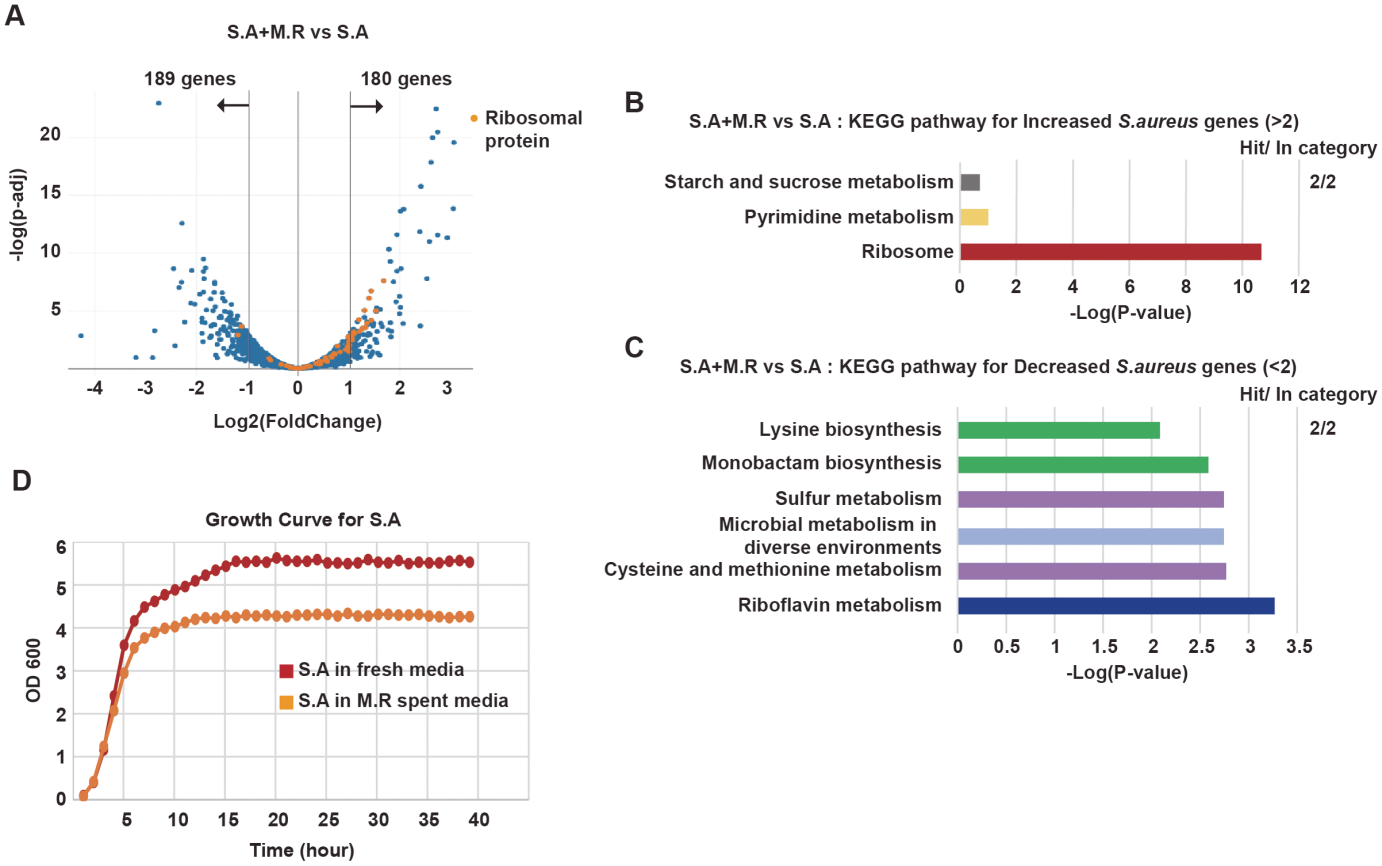
Transcriptional changes in *S. aureus* upon co-culturing with *M. restricta*. **A**. Volcano plot showing transcriptional changes in *S. aureus* co-cultured with *M. restricta* compared to *S. aureus* axenic culture (S.A+M.R vs S.A). **B**. KEGG pathway groups for *S. aureus* genes exhibiting more than 2-fold increase (S.A+M.R vs S.A). **C**. KEGG pathway groups for *S. aureus* genes exhibiting more than 2-fold decrease (S.A+M.R vs S.A). **D**. Growth curve of *S. aureus* in fresh or *M. restricta* spent media.

To comprehensively explore the interplay between *Malassezia* and *Staphylococcus*, we also examined the global changes in gene expression in *Staphylococcus* species after co-culturing with *Malassezia*. In the analysis of bacterial RNA-seq, 180 and 189 genes were found to be significantly upregulated and downregulated, respectively, in *S. aureus* co-cultured with *M. restricta* (Fig. 6A). Notably, RPGs in *S. aureus* were drastically upregulated, as indicated by GO analysis with KEGG pathway (Figs. 6A and 6B). On the contrary, genes involved in riboflavin metabolism and sulfur metabolism were significantly downregulated upon co-culturing with *M. restricta* (Fig. 6C). To understand the physiological changes in *S. aureus* induced by *M. restricta*, we examined the growth rate of *S. aureus* in *M. restricta* spent medium. We observed a decrease in the growth rate of *S. aureus* in the spent medium (OD600: ~4), compared to cultivation in fresh media (OD600: 5.5) (Fig. 6D). We noticed that the decrease in bacterial growth rates upon culturing in *M. restricta* spent medium was specific to *S. aureus* since the growth rate of *S. epidermidis* was not reduced in *M. restricta* spent medium (Fig. S4). Under these stressful conditions, *S. aureus* may express essential genes for cell viability, such as RPGs. It has been known that riboflavin is a precursor for essential co-enzymes FAD and FMN. Therefore, downregulation of genes involved in riboflavin metabolism might be one of the reasons for the decreased growth rate.

## Discussion

**Fig. 7:**
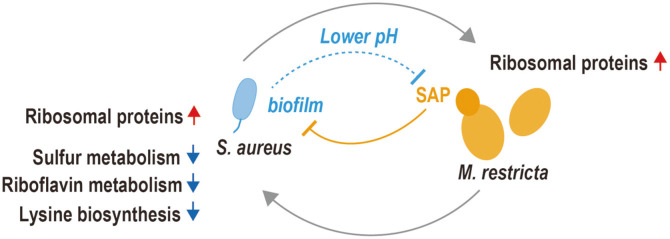
Schematic model of the interplay between *M. restricta* and *S. aureus*. See the discussion for details.

In the present study, we explored the inter-kingdom interactions between two major microorganisms present in skin microbiome, the fungal species *M. restricta* and the bacterial *Staphylococcus* species. The interplay between the two organisms under co-culturing conditions are schematically summarized in Fig. 7. In *M. restricta*, RPGs were upregulated, and putative SAP genes were downregulated upon co-culturing with *Staphylococcus* species. In cells growing under favorable conditions, a great portion of total cellular energy is dedicated to ribosomal biogenesis [20-22]. Tight control of RPG expression is necessary in response to changes in nutrient and energy supply. Transcriptional regulation of RPGs is controlled via the target of rapamycin (TOR) pathway in response to nutrient starvation and other signals. When cells are exposed to nutrient starvation, TORC1 is inhibited, and downstream transcriptional regulators repress transcription of RPGs [20-22]. Thus, we assume that *M. restricta* co-cultured with *S. aureus* may experience favorable growing conditions or may be responding to other uncharacterized signals. The growth rate of *M. restricta* cultured in *S. aureus* spent media was similar to that observed in fresh media, but the growth rate of *M. restricta* cultured in *S. epidermidis* spent media was significantly lower than the growth rate observed in fresh media. Furthermore, we observed differential regulation of RPGs in *M. restricta* according to sub-cellular localization, motif sequences on the promoters of RPGs, and co-cultured *Staphylococcus* species.

Genes encoding putative SAPs, MRET_3770, MRET_2827, and MRET_4390, were drastically downregulated in *M. restricta* co-cultured with either *Staphylococcus* species. However, these three genes were not homologs of the SAP genes previously characterized in *M. globosa* and *M. furfur* [18, 19]. In the present work, we first identified the putative SAPs in *M. restricta* using the concentrated extracellular media by pepstatin A-agarose resin, which is a reversible aspartyl protease inhibitor [19]. We collected proteins specifically bound to pepstatin A-agarose and performed mass spectrometry. Then, we identified MRET_3770 and MRET_2827 as major pepstatin A binding proteins present in the extracellular medium. Further analyses might be required to examine the biochemical activities of aspartyl proteases as in the cases of *M. globosa* and *M. furfur* [18, 19].

We asked how these putative SAPs in *M. restricta* were downregulated in co-cultured conditions. We noticed that the pH of the co-culture medium was reduced from 6.7 to 5–5.5, presumably due to the organic acids emitted by *Staphylococcus* as a by-product during growth [23] and found that the reduced pH induces the downregulation of putative SAPs, such as MRET_3770, MRET_2827, and MRET_4390. Therefore, we hypothesized that *Staphylococcus* species protect the biofilms they form by inhibiting the predominant SAPs by reducing the pH. We further examined the expression of MGL_1932, a previously characterized SAP in *M. globosa* (MGSAP1) by lower pH or co-culturing with *Staphylococcus* species [19]. The reduced pH or co-culturing did not affect the transcriptional regulation of MGL_1932, but instead resulted in the downregulation of MGL_3328, the SAP with the highest expression levels in *M. globosa*. The protein encoded by MGL_3328 might be another functional SAP candidate in *M. globosa*. Recent interesting study reported that MGSAP1 was upregulated in atopic and seborrheic dermatitis skin. Furthermore, this SAP play a pivotal role in fungal skin colonization and tissue inflammation in diseased skin tissue [24]. In our present work, pH is another factor which could control the expression of SAP. Thus, it will be interesting to see the environmental factors that affect the expression of SAP in the diseased skin tissues.

To comprehensively understand the transcriptional interplay between *M. restricta* and *Staphylococcus* species, we also performed bacterial RNA-seq of *S. aureus* co-cultured with *M. restricta*. We observed that RPGs were upregulated in co-cultured *S. aureus*. Regulation of bacterial RPGs have been not intensively studied but is known to be linked to a stress response mechanism referred to as “the stringent response”. The expression of RPGs might be repressed as a part of the stringent response by the function of ppGpp. In addition, ppGpp and the transcription factor DksA bind to the promoter regions of RPGs and inhibit transcriptional initiation in *Escherichia coli* [25, 26]. In our experiment, the co-culture conditions might be unfavorable for *S. aureus* since the growth rate of *S. aureus* in *M. restricta* spent medium was retarded, but the expression levels of RPGs were significantly elevated. Our data suggest that *S. aureus* may have an unknown mechanism to upregulate the expression of RPGs in response to unfavorable growth conditions. Co-cultured *S. aureus* exhibited downregulated expression of genes involved in riboflavin biosynthesis. Riboflavin is a precursor of co-enzymes FAD and FMN, which function as the prosthetic groups of various oxidoreductases. Bacterial riboflavin homeostasis is regulated by FMN riboswitches [27-31]. FMN incorporation into its cognate riboswitch inhibit proper transcription of *rib* genes. Thus, cellular concentration of FMN is critical for controlling the riboswitch, and the FMN riboswitches are among the most sensitive regulators of cellular homeostasis in response to environmental or cellular stressors [27]. Riboflavin is synthesized in bacteria, yeast, and plants, but vertebrates acquire riboflavin from their diet [32]. *S. aureus* obtains riboflavin from the host environment or generates it through *de novo* biosynthesis [28, 33]. Therefore, the downregulation of riboflavin biosynthesis genes might be caused by the riboflavin available in the co-culture environment. *M. restricta* may produce and secret riboflavin or FMN, which would in turn repress bacterial FMN riboswitches, and the bacteria may use FMN acquired from co-culture environment. It is possible that *M. restricta* and *Staphylococcus* species may compete for riboflavin uptake in the co-culture environment.

Collectively, we have explored the interplay between *M. restricta* and *Staphylococcus* species, which co-exist in human skin microbiome. We observed the upregulation of genes encoding ribosomal proteins and the downregulation of genes encoding SAP in co-cultured *M. restricta*. Then, we noticed that the lower pH is critical factor to control the transcriptional expression of SAP coding genes in *M. restricta*. We also observed the upregulation of genes encoding ribosomal proteins and downregulation of genes involved in riboflavin metabolism in co-cultured *S. aureus*. Therefore, we found that the dominant fungus *M. restricta* and the bacteria *Staphylococcus* species interact with each other to compete or reconcile in a given co-existing environment.

## Supplemental Materials

Supplementary data for this paper are available on-line only at http://jmb.or.kr.

## Figures and Tables

**Fig. 3:**
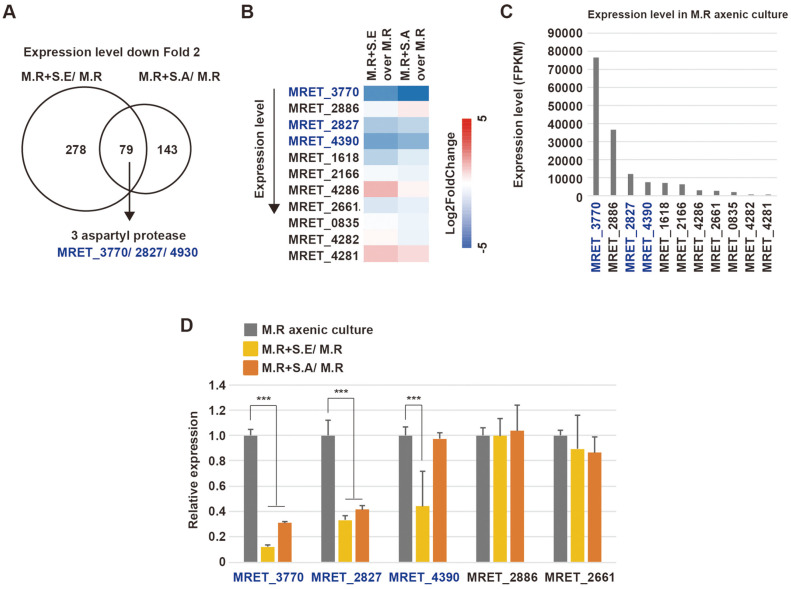
Downregulation of aspartyl protease-encoding genes in *M. restricta* upon co-culturing with *Staphylococcus* spp. **A**. Venn diagram showing the numbers of *M. restricta* (M.R) genes that were downregulated at least 2- fold in both co-culture conditions compared to axenic culture. **B**. Expressional changes in genes encoding aspartyl proteases upon co-culturing. Aspartyl protease-encoding genes were ordered by expression level from top to bottom. **C**. Expression levels of genes encoding aspartyl proteases in *M. restricta* (M.R) axenic culture. **D**. Relative expression levels of genes encoding aspartyl proteases in *M. restricta* (M.R) axenic culture, in *M. restricta* co-cultured with *S. epidermidis* compared to *M. restricta* axenic culture (M.R+S.E/ M.R), and in *M. restricta* co-cultured with *S. aureus* compared to *M. restricta* axenic culture (M.R+S.A/ M.R), examined by reverse transcription followed by qPCR. Expression levels were normalized to those of actin (MRET_1518) as an endogenous control and compared to the expression levels observed in M.R axenic culture. *, *p* < 0.1; **, *p* < 0.01; ***, *p* < 0.001.
