# Supplementary material for: Transcriptional Interplay between Malassezia restricta and Staphylococcus Species Co-Existing in the Skin Environment
Source: J Microbiol Biotechnol. 2023 Jan 6;33(3):319–28. doi: 10.4014/jmb.2212.12026 (PMC10084760; doi:10.4014/jmb.2212.12026)
Supplement: Supplementary file 1 [file jmb-33-3-319-supple.pdf]

J. Microbiol. Biotechnol.
https://doi.org/10.4014/jmb.2212.12026

Supplementary Information

Supplementary Figures

Supplemental Figure1

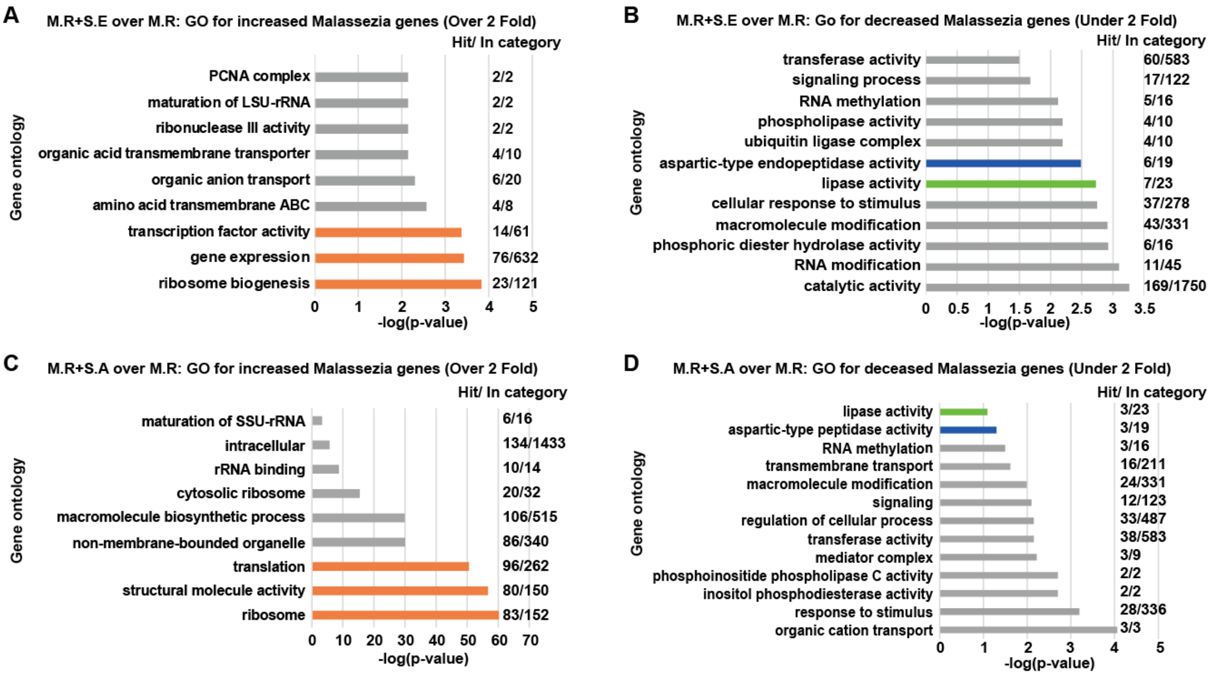

Supplementary Figure 1. Gene ontology analyses for genes changed more than twice.

A: Gene ontology for increased *M. restricta* genes at co-culture with *S. epidermidis*.

B: Gene ontology for decreased *M. restricta* genes at co-culture with *S. epidermidis*.

C: Gene ontology for increased *M. restricta* genes at co-culture with *S. aureus*.

D: Gene ontology for decreased *M. restricta* genes at co-culture with *S. aureus*.

Supplemental Figure 2

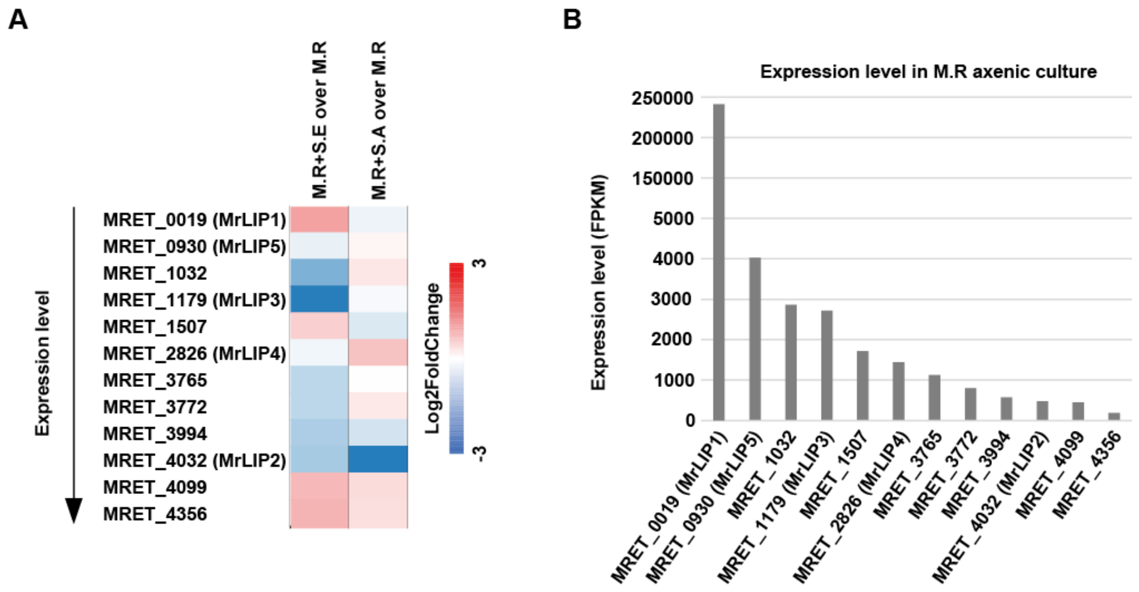

Supplementary Figure 2. Transcriptional changes of lipase coding genes in *M. restricta* by co-culture with *Staphylococcus* spp.

A: Expressional changes for genes encoding lipase at co-cultured conditions. Lipase coding genes were ordered from top to bottom on the basis of expression level.

B: Expression level of genes encoding lipase in *M. restricta* (M.R) axenic culture.

Supplemental Figure3

A

type	number	SignalP
Aspartic (A)	13	11
Cysteine (C)	36	0
Glutamic (G)	0	0
Metallo (M)	40	4
Asparagine (N)	0	0
Mixed (P)	0	0
Serine (S)	30	7
Threonine (T)	16	1
Unknown (U)	0	0
total	135	23

MRET_0835
MRET_1618
MRET_2166
MRET_2661
MRET_2827
MRET_2886
MRET_3770
MRET_4281
MRET_4282
MRET_4286
MRET_4390

B

MRET_Gene	product	Coverage [%]	# Peptides	# PSMs	# Unique Peptides	# AAs	MW [kDa]	Score Sequest HT
MRET_3770	Secreted aspartyl protease	36	15	33	11	375	42	95.82
MRET_2827	Secreted aspartyl protease	43	16	25	16	396	42.5	70.2
MRET_2825	Secreted aspartyl protease	23	9	19	5	375	42.4	54.18
MRET_2661	Aspartic endopeptidase	18	9	15	9	392	42.8	42.78

Supplementary Figure 3. Prediction of secretory aspartyl protease and the result of mass-spectrometry.

A: Prediction for type of proteases in *M. restricta* using MEROPS (https://www.ebi.ac.uk/merops/). And number of secretory proteases predicted by SignalP5.0. Aspartic type proteases are shown in red color.

B: Result of Liquid chromatography-tandem mass spectrometry (LC-MS/MS) for putative aspartyl proteases. LC-MS/MS was conducted by the Proteinworks (www.proteinworks.co.kr).

Supplemental Figure4

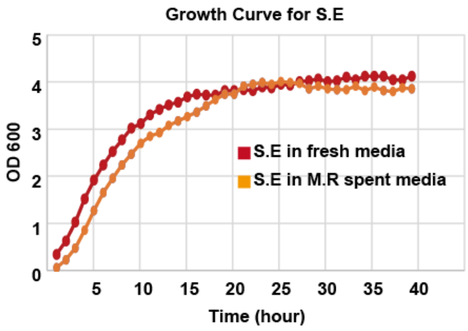

Supplementary Figure 4. Growth curve of *S. epidermidis* in fresh or *M. restricta* spent media.
